# Diagnostic and therapeutic practices for paediatric malaria: Gap between guidelines and clinical reality in south-east Gabon

**DOI:** 10.5281/zenodo.20271870

**Published:** 2026-05-18

**Authors:** Jean-Claude Biteghe-Bi-Essone, Roméo-Karl Imboumy-Limoukou, Ingrid-Nacimiento D’Alva Noronha, Diamella-Nancy Moukodoum, Steede-Seinnat Ontoua, Nick Atiga, Lady Charlène Kouna, Alain-Prince Okouga, Jean Bernard Lekana-Douki, Lydie Sandrine Oyegue-Liabagui

**Affiliations:** 1 Unit of Evolution, Epidemiology and Parasitic Resistances (UNEEREP), Centre Interdisciplinaire de Recherches Médicales de Franceville (CIRMF), B.P. 769 Franceville, Gabon.; 2 Department of Parasitology-Mycology, Université des Sciences de la Santé, BP 4008, Libreville, Gabon, BP 4008, Libreville, Gabon.; 3 Ecole Doctoral régional en Infectiologie Tropical, Franceville, Gabon.; 4 Laboratory of Molecular and Cellular Biology (LABMC), Université des Sciences et Techniques de Masuku Franceville, Gabon.

## Abstract

**Background:**

Malaria is highly prevalent in Gabon. The objective of this study was to assess adherence to WHO recommendations in the management of paediatric malaria in Franceville, south-east Gabon, and to identify factors influencing clinical decision making.

**Materials and Methods:**

This was a prospective observational study conducted from January 2021 to July 2023. Information regarding clinical examination, thick blood smear results, and the prescription of antimalarial drugs of paediatric children were collected anonymously following informed consent from parents or legal guardians. Data were analysed using R software (version 4.0.5), with descriptive statistics, Chi-square tests, and binary logistic regression to estimate crude and adjusted odds ratios with 95% confidence intervals at a significance level of p < 0.05.

**Results:**

A total of 3,195 children aged 0-16 years presenting with fever or a history of fever were included. Overall, 41.6% of patients tested positive by thick blood smear, and 21.4% of them had severe malaria. Despite negative thick blood smear results, 86.7% of patients received a presumptive diagnosis and 98.8% of them were treated with antimalarials. Symptoms significantly associated with a presumptive diagnosis were asthenia (aOR = 1.41; 95% CI [1.01–2.00]), headache (aOR = 1.71; 95% CI [1.02–3.03]), and nausea-vomiting (aOR = 1.95, 95% CI [1.20–3.31]).

**Conclusions:**

Despite access to parasitological testing, presumptive diagnosis remains common, exposing patients to potential overprescription and increased risk of drug resistance. Strengthening diagnostic capacity and continuous training of healthcare providers are essential to improve case management.

## Introduction

Malaria remains a major public health concern, particularly in sub-Saharan Africa. Children under five years of age and pregnant women are the most vulnerable populations [1]. The high lethality associated with certain severe forms of the disease [2] often leads healthcare providers to suspect malaria in any patient presenting with fever, regardless of accompanying clinical signs, especially in endemic areas [1].

The global malaria control strategy relies primarily on early detection of cases and prompt administration of appropriate treatment [1]. In general, malaria diagnosis is based on clinical examination, which should be complemented by the examination of thick and thin blood smears under microscopy or by rapid diagnostic tests (RDTs) to confirm the presence or absence of *Plasmodium* parasites in the blood. Accurate malaria diagnosis thus remains a major challenge in endemic regions. Indeed, non-specific symptoms associated with malaria, such as fever, asthenia, or digestive disorders, frequently overlap with those of other common tropical diseases [3, 4]. This symptom overlap, combined with the lack of highly sensitive diagnostic tools such as polymerase chain reaction (PCR) in most health facilities, often leads to presumptive diagnoses and empirical treatments [5]. Studies have reported that antimalarial drugs are still prescribed despite negative microscopy or RDT results [3,4,6], which contradicts World Health Organization (WHO) recommendations stating that when parasitological testing is available, antimalarials should only be prescribed to patients with positive test results [1].

As in all sub-Saharan African countries, malaria is endemic in Gabon, with year-round transmission sustained by climatic conditions favourable to the development of *Anopheles* vectors [7]. To address this, the country has adopted WHO recommendations to systematically perform an RDT and/or thick blood smear in all suspected cases before initiating treatment. However, no study has yet assessed compliance with these WHO guidelines in Gabon, although several studies have documented antimalarial prescribing practices in the subregion [4,8,9]. This study therefore aimed to analyse the diagnostic and therapeutic practices for malaria in paediatric settings in Franceville, with particular focus on evaluating the alignment between national protocols and their implementation in routine practice, as well as identifying factors influencing clinical decision making.

## Materials and Methods

This study was conducted in the paediatric department of the Sino-Gabonese Friendship Hospital (HASG) in Franceville, between 1 January 2021 and 31 July 2023. The HASG, which is the second-largest hospital facility in the city, plays a central role in providing healthcare services to the local population. Franceville is an urban agglomeration located in the Haut-Ogooué province of Gabon. It ranks as the second-largest city in Gabon in terms of population, with an estimated number of inhabitants of 129,694 [10]. In this region, the primary vectors of malaria transmission are *Anopheles funestus* and *An. gambiae s.l.*. Severe cases of malaria, particularly those caused by *Plasmodium falciparum*, peak during the rainy season [2]. This study included all patients aged 0-16 years that were admitted to and hospitalised in the paediatric department.

### Procedures for data collection and clinical diagnosis of malaria

This cross-sectional study investigated the management practices of febrile patients in a high malaria-endemic setting. Data were extracted from the medical records of febrile patients who consulted at HASG during the study period. All clinical, anthropometric, and demographic information recorded at the time of consultation was included. Clinical diagnoses established by physicians, results of laboratory investigations performed by hospital laboratory technicians upon medical request, reported symptoms, treatments administered, and any hospital admissions were systematically reviewed. The clinical diagnosis of malaria, as documented in the medical records, was based on the signs and symptoms reported by patients and the physical findings observed by healthcare providers during clinical examination [11].

### Laboratory diagnosis of malaria

All laboratory work was conducted at the HASG laboratory by trained laboratory technicians. *Plasmodium* spp. infection was detected using the thick blood smear method, which involved placing a drop of blood at the centre of a microscope slide. The slide was air-dried and then stained with 20% Giemsa for 15 min before being examined under a microscope. Thick blood smears were classified as positive when asexual stages of *Plasmodium* spp. were identified by two independent microscopists. Parasite density was quantified following WHO criteria [12]: low parasitaemia (+) corresponded to 1–10 parasites per 100 fields, mild (++) to 11–100 parasites per 100 fields, moderate (+++) to 1–10 parasites per single field, and high parasitaemia (+ +++) to more than 10 parasites per single field.

### Operational definitions

Febrile malaria was defined as an individual presenting with fever (axillary temperature ≥ 37.5 °C) or reporting a febrile episode within the 48 hrs preceding the examination, associated with a positive thick blood smear [13]. Presumptive malaria diagnosis (PMD) were patients that exhibited symptoms suggestive of malaria but lacked confirmation by a positive thick blood smear.

### Ethical considerations

This study received approval from the Ministry of Health, the National Research Ethics Committee of Gabon (Reference No. 0034/2020/CNER/P/SG), and the South-East Regional Health Directorate of the Gabonese Republic (Reference No. 1784/MS/SG/DRSSE). Participation was entirely voluntary, contingent upon obtaining informed consent, and all reported results were handled with strict confidentiality.

### Data analysis

Data from all patients were recorded in Excel 2013 spreadsheets. Statistical analyses were performed using R software version 4.0.5. Qualitative variables were described using proportions, while quantitative variables were summarised using the mean, standard deviation (SD), median, and interquartile range (IQR). Proportions were compared using the Chi-square test. For all analyses, the significance level was set at α = 5%. A binary logistic regression model was employed to estimate adjusted odds ratios (aOR) for the independent risk factors. Crude odds ratios (OR) were also reported. A 95% confidence interval (95% CI) was applied, and statistical significance was determined at a threshold of α = 0.05.

## Results

### Demographic, anthropometric, and clinical characteristics of patients

A total of 3,195 patients were included in the study; 1,570 were male and 1,625 were female, yielding a male-to-female sex ratio of 0.97. The mean age (±SD) of the patients was 4.61 ± 4.09 years. The age distribution (Table 1) shows that the majority of patients (66.51%) were aged 0–5 yrs, while 23.82% were 6-11 yrs old, and 9.67% were 12 yrs or older.

**Table 1:** Socio-demographic, anthropometric and clinical characteristics of patients (n=3195).

Parameter	Number
Gender	
Male	1570
Female	1625
Age (years)	
Mean (SD)	4.61 (4.09)
Median [IQR]	3.0 [1 - 7]
Weight (in kg)	
Mean (SD)	18.4 (12.3)
Median [IQR]	14.5 [10 - 22]
Age groups (years)	
0 – 5, n (%)	2125 (66.51)
6 – 11, n (%)	761 (23.82)
≥ 12, n (%)	309 (9.67)
Temperature (in °C)	
Mean (SD)	37.1 (0.98)
Median [IQR]	36.8 [36.4 - 37.6]

The mean (±SD) body weight of patients was 18.38 ± 12.3 kg. The mean (±SD) body temperature and haemoglobin level were 37.1±0.98 °C and 10.1 ± 1.97 g/dL, respectively (median haemoglobin level: 10.2 g/dL, IQR = [9.10–11.3]).

### Reasons for consultation

Besides fever or a history of fever, the main additional reasons for consultation are shown in Table 2.

**Table 2:** Prevalence of clinical symptoms in patients (n=3195).

Symptom	n (%)
Cough	1679 (52.53)
Rhinorrhoea	1226 (38.36)
Asthenia	1139 (35.64)
Anorexia	892 (27.91)
Nausea-vomiting	589 (18.43)
Headache	523 (16.36)
Diarrhoea	391 (12.23)
Abdominal pain	339 (10.61)
Articular pains	172 (5.38)
Other symptoms*	212 (2.91)

* All less frequent symptoms not specifically linked to malaria (see Appendix 1)

The main symptoms accompanying the fever were cough, followed by rhinorrhoea, asthenia, and anorexia. Other less frequent symptoms included vomiting, headache, diarrhoea, abdominal pain, and polyarthralgia.

### Paraclinical diagnosis of malaria

Among the 3,195 febrile patients, 41.56% (n = 1,328; 95% CI [39.87–43.28]) were diagnosed with malaria, while 58.44% (n = 1,867) had a negative thick blood smear. The analysis revealed several factors significantly associated with malaria infection (Table 3). On average (±SD), patients with malaria were older than non-infected patients (61.4 ± 50 vs. 53.1 ± 45.9 months; p < 0.001). However, when considering age-group distribution, children aged 0–5 yrs accounted for the highest proportion of malaria cases (p < 0.001). Clinically, symptoms such as asthenia (p < 0.001), headache (p < 0.001), abdominal pain (p = 0.046), and vomiting (p < 0.001) were significantly more common among malaria-positive patients. In contrast, rhinorrhoea (p < 0.001) and cough (p < 0.001) were significantly less prevalent in this group. Biologically, the mean (±SD) haemoglobin level was significantly lower (p < 0.001) in infected patients (9.79 ± 2.27 g/dL) compared to non-infected patients (10.3 ± 1.70 g/dL).

**Table 3:** Clinical, biological and anthropometric parameters associated with malaria.

		n	Malaria infection, n (%)		*p*-value
			No	Yes	
Total		3195	1867	1328	
Gender	Female	1625	959 (51)	666 (50)	0.5
	Male	1570	908 (49)	662 (50)	
Age groups (years)	(0-5)	2125	1302 (70)	823 (62)	< 0.001
	(6-11)	761	416 (22)	345 (26)	
	≥ 12	309	149 (8)	160 (12)	
Asthenia	No	2056	1267 (68)	789 (59)	< 0.001
	Yes	1139	600 (32)	539 (41)	
Headache	No	2672	1607 (86)	1065 (80)	< 0.001
	Yes	523	260 (14)	263 (20)	
Abdominal pain	No	2856	1686 (90)	1170 (88)	0.046
	Yes	339	181 (10)	158 (12)	
Rhinorrhea	No	1969	1060 (57)	909 (68)	< 0.001
	Yes	1226	807 (43)	419 (32)	
Cough	Yes	1679	1061 (57)	618 (47)	< 0.001
	No	1516	806 (43)	710 (53)	
Nausea-vomiting	No	2606	1559 (84)	1047 (79)	< 0.001
	Yes	589	308 (16)	281 (21)	
Anorexia	No	2303	1339 (72)	964 (73)	0.59
	Yes	892	528 (28)	364 (27)	
Diarrhoea	No	2804	1650 (88)	1154 (87)	0.21
	Yes	391	217 (12)	174 (13)	
Articular pains	No	3023	1791 (96)	1232 (93)	< 0.001
	Yes	172	76 (4)	96 (7)	
Other symptoms*	No	2983	1755 (94)	1228 (92)	0.09
	Yes	212	112 (6)	100 (8)	

* See Appendix 1

### Severe malaria

Among the 1,328 patients diagnosed with malaria, 21.39% (n = 284; 95% CI [19.26–23.67]) had severe malaria (Table 4). The main severity criteria observed in these patients were severe anaemia (48.23%, n = 137), hyperparasitaemia (22.89%, n = 65), and convulsions (14.79%, n = 42). The most common co-morbidities were bronchitis (40%), nasopharyngitis (20%), and gastrointestinal infections (10%).

**Table 4:** Prevalence of severity criteria in severe malaria patients.

Severity criteria	n (%)
Severe anaemia	137 (48.23)
Convulsion	42 (14.79)
Respiratory distress	22 (7.75)
Hyperparasitaemia	65 (22.89)
Prostration	17 (5.99)

### Presumptive Diagnosis of Malaria and Associated Factors

Among the 1,867 patients with negative thick blood smear results, PMD was established in 86.66% of cases (n = 1,618; 95% CI [85.05–88.13]). Several factors were associated with presumptive malaria diagnosis (Table 5).

**Table 5:** Clinical, biological and anthropometric parameters associated with the presumptive diagnosis (PMD) of malaria.

		n	PMD, n (%)		*p* value
			Yes	No	
Total		1867	1618	249	
Gender	Female		829 (51)	130 (52)	0.77
	Male		789 (49)	119 (48)	
Age groups (years)	0 – 5	1302	1127 (70)	175 (70)	0.44
	6 – 11	416	366 (23)	50 (20)	
	≥ 12	149	125 (7.7)	24 (9.6)	
Asthenia	No	1267	1073 (66)	194 (78)	< 0.001
	Yes	600	545 (34)	55 (22)	
Headache	No	1607	1376 (85)	231 (93)	< 0.01
	Yes	260	242 (15)	18 (7.2)	
Abdominal pain	Non	1686	1449 (90)	237 (95)	< 0.01
	Yes	181	169 (10)	12 (4.8)	
Rhinorrhea	No	1060	939 (58)	121 (49)	< 0.01
	Yes	807	679 (42)	128 (51)	
Cough	Yes	1061	903 (56)	158 (63)	0.02
	No	806	715 (44)	91 (37)	
Anorexia	No	1339	1146 (71)	193 (78)	0.03
	Yes	528	472 (29)	56 (22)	
Nausea-vomiting	No	1559	1330 (82)	229 (92)	< 0.001
	Yes	308	288 (18)	20 (8)	
Articular pains	No	1791	1548 (96)	243 (98)	0.15
	Yes	76	70 (4)	6 (2)	
Diarrhoea	No	1650	1433 (89)	217 (87)	0.52
	Yes	217	185 (11)	32 (13)	
Other symptoms*	No	1755	1541 (95)	214 (86)	< 0.001
	Yes	112	77 (5)	35 (14)	

* See Appendix 1

Patients with a PMD were, on average (±SD), younger (53.2 ± 45.7 months) than those without this diagnosis (60 ± 49.7 months; p < 0.001). A higher proportion of presumptive malaria cases was observed among children aged 0-5 years (p < 0.001). Clinical symptoms such as asthenia (p = 0.019), headache (p = 0.029), and cough (p < 0.001) were more frequent among patients diagnosed with suspected malaria. In contrast, rhinorrhoea was less common in this group (p < 0.001).

Patients with a PMD were, on average (±SD), younger (53.2 ± 45.7 months) than those without this diagnosis (60 ± 49.7 months; p < 0.001). A higher proportion of presumptive malaria cases was observed among children aged 0-5 years (p < 0.001). Clinical symptoms such as asthenia (p < 0.001), headache (p < 0.01), and cough (p = 0.02) were significantly more frequent among patients diagnosed with suspected malaria. In contrast, rhinorrhoea was less common in this group (p < 0.001).

### Multivariate Analysis of Associated Risk Factors

Table 6 presents clinical symptoms that may be interpreted as indicative of probable malaria despite a negative thick blood smear. Multivariate analysis revealed that the presence of asthenia (aOR = 1.41; 95% CI: 1.01–2.00; p = 0.05), headache (aOR = 1.71; 95% CI: 1.02–3.03; p = 0.05), and vomiting (aOR = 1.95; 95% CI: 1.20–3.31; p < 0.01) significantly increased the likelihood of a PMD, even in the absence of a positive thick blood smear. Conversely, the presence of clinical signs unlikely to be associated or confused with malaria significantly reduced this risk (aOR = 0.35; 95% CI: 0.19–0.65; p < 0.001). Additionally, symptoms such as rhinorrhoea, cough, anorexia, and polyarthralgia, as well as body temperature, were not significantly associated with presumptive malaria (p > 0.05).

**Table 6:** Multivariate analysis of risk factors associated with probable malaria in patients with negative parasitological test.

	n	PMD, n (%)	Univariate	Multivariate	p-value
			OR (95%CI)	aOR (95% CI)	
Total	1867	-	-	-	-
Asthenia					
No	1267	1073 (84.69)	1	1	Ref
Yes	600	545 (90.83)	1.79 (1.31-2.46)	1.41 (1.01-2.00)	0.05
Headache					
No	1607	1376 (85.63)	1	1	Ref
Yes	260	242 (93.08)	2.26 (1.37-3.71)	1.71 (1.02-3.03)	0.05
Rhinorrhea					
No	1060	939 (88.58)	1	1	Ref
Yes	807	679 (84.14)	0.68 (0.52-0.89)	0.82 (0.58-1.15)	0.25
Cough					
No	1061	903 (85.11)	1	1	Ref
Yes	806	715 (88.71)	1.37 (1.04-1.81)	1.23 (0.84-1.83)	0.29
Nausea-vomiting					
No	1559	1330 (85.31)	1	1	Ref
Yes	308	288 (93.51)	2.48 (1.54-3.98)	1.95 (1.20-3.31)	< 0.01
Anorexia					
No	1339	1146 (85.59)	1	1	Ref
Yes	528	472 (89.39)	1.42 (1.03-1.94)	1.27 (0.90-1.80)	0.17
Articular pains					
No	1791	1548 (86.43)	1	1	Ref
Yes	76	70 (92.11)	1.83 (0.79-4.26)	1.18 (0.52-3.14)	0.72
Other symptoms*					
No	1775	1541 (86.82)	1	1	Ref
Yes	112	77 (68.75)	0.33 (0.22-0.51)	0.35 (0.19-0.65)	< 0.001

### Treatment practices

Overall, 80.81% of patients (n = 2582) did not engage in self-medication prior to medical consultation, whereas 19.19% (n = 613) reported using medications without a medical prescription. Patients diagnosed with malaria were treated either with artemether-lumefantrine combination therapy (53.69%, n = 713) or with artesunate injection (46.31%, n = 615) for those unable to swallow tablets or who had vomited following oral administration of the combination therapy. Among the 1618 patients with a PMD, 98.76% (n = 1598) received antimalarial treatment: 85.05% were treated with artemether-lumefantrine combination, 0.74% (n = 12) with dihydroartemisinin alone, and 14.21% (n = 227) with artesunate injection. Only 1.23% (n = 20) did not receive any antimalarial treatment.

## Discussion

This study evaluated the management practices of febrile patients suspected of malaria at the paediatric department of the Sino-Gabonese Friendship Hospital in Franceville, located in southeastern Gabon. The results revealed a high prevalence of malaria among febrile children, estimated at 41.56% (95% CI: 39.87–43.28). These findings confirm, on one hand, the major role of malaria in the aetiology of febrile syndromes in Gabon [13, 14], and on the other hand, suggest a sustained or even increasing prevalence despite the end of the COVID-19 pandemic [15]. This situation may be explained by insufficient investment in malaria control measures, even after the pandemic, which had been a primary cause of underfunding these efforts [16].

The symptoms most frequently associated with malaria (asthenia, headache, nausea, and vomiting) correspond to the classic manifestations of *P. falciparum* malaria [13, 17]. This observation aligns with epidemiological data from Gabon, where *P. falciparum* accounts for 90-100% of malaria cases [13,14,18], confirming its dominant role in the clinical presentation. Among malaria patients, 284 (21.39%) presented with severe malaria, with severe anaemia being the most common complication (48.23%, n = 137), followed by hyperparasitaemia (22.89%, n = 65) and seizures (14.79%, n = 42). These findings are consistent with previous studies conducted in Gabon, which identify these criteria as the main manifestations of severe malaria in the country [19, 20]. The predominance of severe anaemia may reflect local factors such as nutritional deficiencies or co-infections with intestinal parasites, which are common in the Gabonese context [21, 22].

This study revealed a high proportion of presumptive malaria diagnoses (86.66%) among patients with negative thick blood smear results. This finding may indicate clinical over-diagnosis of malaria despite the absence of parasitological confirmation. The tendency to diagnose malaria solely based on symptoms and to initiate antimalarial treatment may contribute to the overuse of antimalarial drugs, thereby promoting the emergence of drug resistance [23, 24].

Multivariate analysis identified several symptoms significantly associated with PMD in febrile children with negative thick blood smears: asthenia (aOR = 1.41), headache (aOR = 1.71), and vomiting (aOR = 1.95). Although nonspecific, these signs appear to strongly influence clinical decision-making, likely due to their well-known association with malaria in the Gabonese context [13]. These results suggest that clinicians still heavily rely on clinical judgment despite negative parasitological tests, which may introduce diagnostic bias, highlighting the urgent need to strengthen healthcare workers’ training to promote management based on reliable biological evidence.

Our study also highlighted a high prescription rate of antimalarials in the absence of biological confirmation. Among children with a presumptive diagnosis, 98.76% received treatment, mainly with artemether-lumefantrine combination therapy (85.05%), followed by artesunate injection (14.21%) or dihydroartemisinin alone (0.74%). This practice contradicts WHO recommendations, which advise treating only parasitology-confirmed cases unless diagnostic tests are unavailable within two hours of patient presentation [25]. Only 1.23% of patients did not receive any antimalarial treatment. Such practice, previously reported in several sub-Saharan African regions [4,26,27], may reflect low confidence in negative diagnostic test results (thick smear and/or rapid diagnostic tests) or a ‘treat cautiously’ strategy motivated by fear of missing an undetected infection.

It is important to note that microscopy and RDTs detect approximately half of malaria infections compared to PCR-based methods [28, 29]. These submicroscopic infections, undetectable by routine methods, may cause febrile syndromes in patients [30]. Antimalarial prescription without biological confirmation may also stem from limited knowledge of fever aetiology and associated symptoms. However, such an approach increases the risk of antimalarial misuse, promotes resistance emergence, and may elevate morbidity and mortality due to non-malarial febrile illnesses [24, 31]. These findings underscore the need for improved healthcare worker awareness on rigorous interpretation of diagnostic tests and strict adherence to WHO recommended treatment protocols.

Our study has several limitations. First, the absence of molecular diagnostic techniques, such as PCR, did not allow for the detection of submicroscopic infections. These undetected cases may account for some false-negative results obtained through microscopy or RDTs, and could, in certain contexts, justify the administration of ACTs. Second, the data were collected from a single hospital facility, which may limit the generalisability of the findings at the national level. Indeed, clinical practices observed in Franceville may not accurately reflect those in other regions of Gabon.

## Figures and Tables

**Appendix 1:**
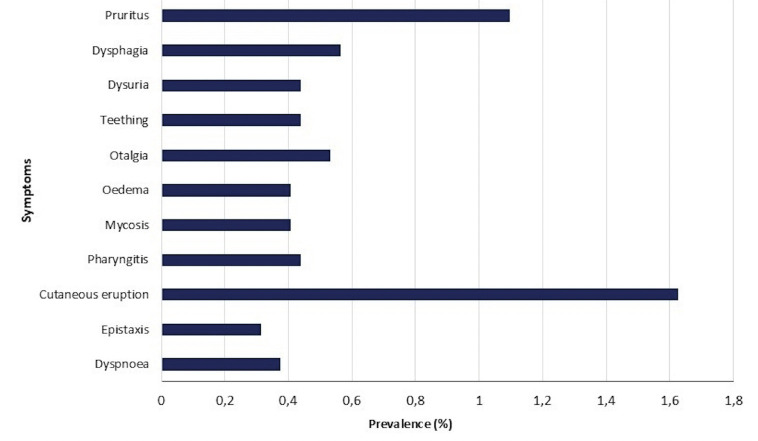
Prevalence of less common symptoms unrelated to malaria.
